# Prone versus supine position for adjuvant breast radiotherapy: a prospective study in patients with pendulous breasts

**DOI:** 10.1186/1748-717X-8-232

**Published:** 2013-10-08

**Authors:** Marco Krengli, Laura Masini, Tina Caltavuturo, Carla Pisani, Giuseppina Apicella, Eleonora Negri, Letizia Deantonio, Marco Brambilla, Giuseppina Gambaro

**Affiliations:** 1 Department of Radiotherapy, University of “Piemonte Orientale”, Via Solaroli 17, 28100 Novara, Italy; 2 Medical Physics, University Hospital “Maggiore della Carità”, Novara, Italy; 3 Department of Translational Medicine, University of “Piemonte Orientale”, Novara, Italy

**Keywords:** Breast cancer, Prone setup, Dosimetric analysis

## Abstract

**Purpose:**

To analyze dosimetric parameters of patients receiving adjuvant breast radiotherapy (RT) in the prone versus supine position.

**Methods and materials:**

Forty-one out of 55 patients with pendulous breasts and candidates for adjuvant RT were enrolled in the study after informed consent. They underwent computed tomography (CT)-simulation in both prone and supine position. Target and non target volumes were outlined on CT images. Prescribed dose was 50 Gy delivered by two tangential photon fields followed by 10 Gy electron boost. Target coverage and dose homogeneity to clinical target volume (CTV) and planning target volume (PTV) were assessed by V95, V105 and V107 and dose to lung, heart and left anterior descending coronary artery (LAD) by V5, V10, V20, and mean and maximum dose. Data were analyzed by Student’s t-test.

**Results:**

CTV and PTV coverage was significantly better in supine than in prone position. Lung V5, V10, and V20 were significantly lower in prone than in supine position. Heart V5, V10, V20, and LAD mean and maximum dose, in the 17 patients with left breast tumor, were lower in prone than in supine position, but without statistical significance. Based on treatment planning data and on treatment feasibility, 29/41 patients (70.7%) were treated in prone position. Acute and late toxicities of patients treated in prone and in supine position were not statistically different.

**Conclusion:**

Prone position is a favorable alternative for irradiation of mammary gland in patients with pendulous breasts and in our series was adopted in 71% of the cases.

## Background

The majority of patients with early-stage breast cancer are candidates for breast-conservation therapy followed by whole-breast irradiation (WBI), typically delivered in the supine position. However, supine WBI does have limitations such as lateral dislocation of breast, accentuation of the infra-mammary folds, and inclusion of lung and heart portion in treatment plan. In particular, irradiation after breast-conserving surgery (BCS) in women with large and/or pendulous breasts is a challenge for radiation oncologists. Increased radiation related toxicity and worse cosmetic outcome was found in patients with large breasts and/or increased body mass index (BMI) [1]. Radiation factors identified as potentially causative include increased dose inhomogeneity from medial to lateral separation of the breast and bolus effect on skin, in the infra-mammary folds, where there is increased skin-on-skin contact. In addition, patients may receive increased doses to critical structures such as the heart or lungs owing to the positioning of the breast on the chest wall when the patient lies supine. Prone breast irradiation aims to improve some of the technical limitations associated with treating large and pendulous breasts and it may limit radiation doses to organs at risk such as lung and heart [2-4].

Our institution developed a method for delivering breast three dimensional conformal radiotherapy (3D-CRT) in the prone position to address the technical challenges associated with irradiation of large and pendulous breasts. The goal of this study is to compare dosimetric parameters in prone versus supine position in a cohort of women with pendulous breasts receiving WBI with two tangential fields after conservative surgery.

## Methods

Fifty-five consecutive patients presenting with pendulous breasts were selected for this prospective study after BCS at our institution. Selection of patients was based on the presence of the typical infra-mammary fold in supine position and on the technical feasibility of the simulation procedure considering the gantry diameter of 70 cm of the helical computed tomography (CT)-scan (Prospeed, General Electric, Milwaukee, USA). The indication of regional node irradiation was an exclusion criteria. The informed consent was obtained in all cases.

CT simulation was performed in both prone and supine position with contiguous slices of 5 mm thickness covering the entire thoracic region from the apex of the lung to the diaphragm. In the prone setup, the patient was positioned on the breast board Clear Vue™ (Orbital Therapy, Bedford, USA) with both arms above the head and the hands holding a handlebar to reduce body rotation. The contralateral breast was lifted away from the treated breast and placed on the top of the mattress. The head was turned away from the treated side. At the time of CT simulation, a posterior-anterior setup point was established and 4 leveling markers on the skin two on the midline in the dorsal region, one at the level of the lower aspect of the scapula and one at the level of the mid-axillary line. Supine patient setup was secured by the breast Posiboard™ system (CIVCO, Kalona, USA). Three skin markers, two on the midline in the sternal region and one in the lateral aspect of the breast, were placed for position verification.

The following target and non-target structures were outlined: clinical target volume (CTV), planning target volumes (PTV), ipsilateral lung, heart, and left anterior descending coronary artery (LAD). CTV was defined as the entire breast tissue starting 5 mm below the skin. PTV was obtained by adding 10 mm margin to the CTV, except in the skin direction.

**Figure 1:**
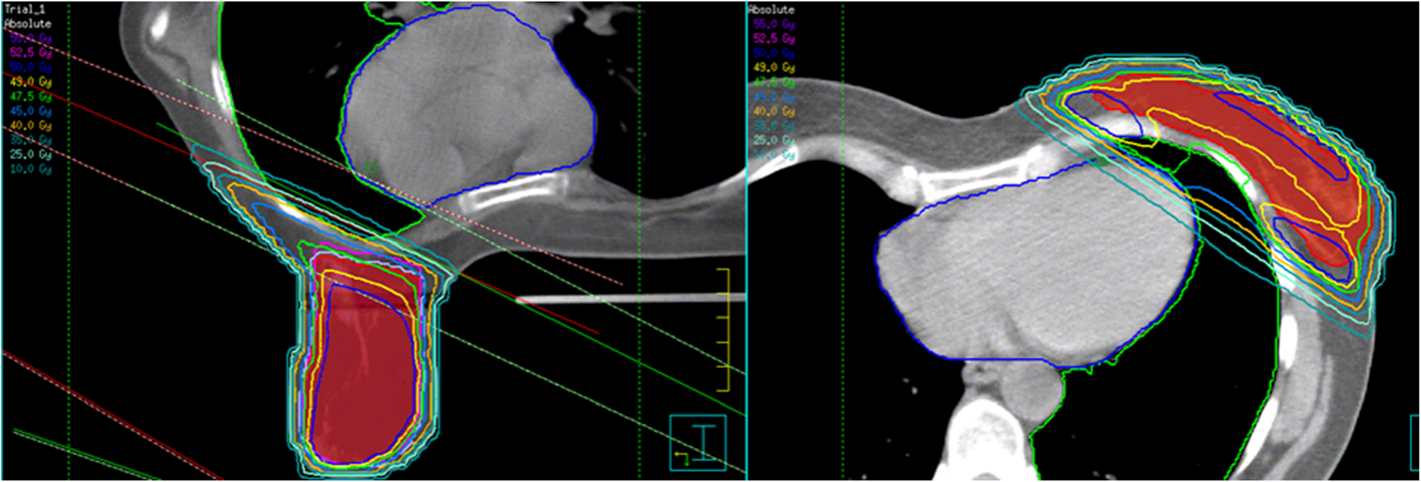
Treatment plans in supine (right) and prone (left) setup.

Treatment technique consisted of two opposed tangential fields by using 6–15 MV photon beams (Figure 1). Radiation fields were appropriately customized by multileaf collimator when needed in order to spare the surrounding healthy tissues. The angle of the beams was adjusted to minimize the irradiation of lung parenchyma and left ventricle. Appropriate physical wedge compensation was used to achieve optimal target coverage and minimize dose heterogeneity. Total dose prescribed was 50 Gy in 25 fractions delivered by the two tangential fields in supine or prone position followed by 10 Gy boost dose in 5 fractions to the tumor bed, delivered by electrons in supine position.

At the first treatment session, portal images of the two tangential treatment fields were acquired and compared with the treatment plan digitally reconstructed radiographs. At the same time, a reference surface image of the thorax was recorded by the AlignRT™ system (VisionRT, London, UK). Surface images were acquired daily during every setup procedure and co-registered with the reference image obtained at the first treatment session.

Dose calculation with a grid of 3 mm was performed using the collapsed cone convolution algorithm of the treatment planning system, including the correction for tissue heterogeneity. Dose volume histograms (DVHs) for CTV, PTV, ipsilateral lung and heart and LAD were calculated.

Two treatment plans were performed for each case in prone and in supine position respectively. Priority was given to target coverage taking into account our dose limitation for lung parenchyma (V20 < 10%) and heart (V5 < 5%) for this specific treatment with two tangential fields.

CTV and PTV coverage was analyzed by using the volume receiving at least 95% (V95), 105% (V105), and 107% (V107) of the prescribed dose. Lung, heart and LAD were analyzed by maximum (Dmax) and mean dose (Dmean), V5, V10, and V20. Statistical analysis was performed with Student’s t-test considering a p value <0.05 as statistically significant. Acute and late toxicity were scored using the Radiation Therapy Oncology Group criteria [5]. Acute toxicity was assessed weekly during treatment and monthly after treatment completion. Late effects were assessed during follow-up visits 6 months after radiotherapy, every 4 months for the first 2 years and every 6 months. The incidence in the two patient groups was analyzed by Fisher’s exact test.

## Results

Forty-one of the 55 (74.5%) selected patients with pendulous breasts were effectively enrolled in our prospective study. All patients presented with infra-mammary fold with potential bolus effect on the skin when laying in supine position. Fourteen patients (25.4%) with larger breasts were excluded because of the insufficient gantry diameter of CT-scan (12 patients, 21.8%) or of the low compliance to prone setup position (2 patients, 3.6%). The main patients characteristics are summarized in Table 1.

**Table 1:** Main patients characteristics

**Characteristics**	**Parameters**	**Values**
Age	Mean	54.9
	Median	55
	Range	30-74
Karnofsky Performance Status	Median	90
	Range	80–100
Body Mass Index	Mean	24.3
	Median	24
	Range	20-32
Breast Side	Right	24 (58.5%)
	Left	17 (41.5%)
Pathological Tumor Stage	Tis	2 (4.9%)
	T1	29 (70.7%)
	T2	10 (24.4%)
Pathological Nodal Stage	N0	28 (68.3%)
	N1	13 (31.7%)
Surgery	Conservative surgery + biopsy of sentinel node	28 (68.3%)
	Conservative surgery + axillary node dissection	13 (31.7%)
Histology	Ductal infiltring carcinoma	31 (75.6%)
	Lobular infiltring carcinoma	5 (12.2%)
	Mixed carcinoma	3 (7.3%)
	In-situ ductal carcinoma	2 (4.9%)
Estrogen/Progesteron Receptors	Positive	27 (65.9%)
	Negative	14 (34.1%)
Anti-Hormone Therapy	Yes	19 (46.3%)
	No	22 (53.7%)
Adjuvant Chemotherapy	Yes	10 (24.4%)
	No	31 (75.6%)
Optimal Position	Prone	29 (70.7%)
	Supine	12 (29.3%)

The results of the dosimetry comparison of the two setup positions for the whole series and for left sided tumors are summarized in Tables 2 and 3.

**Table 2:** Comparison of dosimetric parameters from treatment plans obtained in the two setup positions for the whole patients series (41 patients)

	**Prone setup**	**Supine setup**	**p-value**
PTV (cc)	534.9 ± 229.4	515.3 ± 174.2	0.32
V95 (%)	96.5 ± 3.5	98.0 ± 1.6	0.04
V105 (%)	1.4 ± 1.1	2.0 ± 2.5	0.14
V107 (%)	0.1 ± 0.2	0.2 ± 0.5	0.16
Dmin (Gy)	35.8 ± 11.3	35.8 ± 10.2	0.97
Dmax (Gy)	53.7 ± 0.6	53.7 ± 0.6	0.80
Dmean (Gy)	50.1 ± 0.4	50.1 ± 0.3	0.42
CTV (cc)	468.1 ± 216.6	432.0 ± 159.0	0.02
V95 (%)	98.4 ± 2.3	99.3 ± 1.0	0.02
V105 (%)	1.3 ± 1.1	1.8 ± 2.4	0.25
V107 (%)	0.1 ± 0.2	0.1 ± 0.4	0.45
Dmin (Gy)	42.4 ± 7.4	44.4 ± 4.4	0.15
Dmax (Gy)	53.6 ± 0.6	53.5 ± 0.6	0.42
Dmean (Gy)	50.2 ± 0.4	50.2 ± 0.3	0.92
Lung (cc)	1335.0 ± 331.0	1201.0 ± 264.0	<10^-6^
V20 (%)	1.5 ± 1.8	9.0 ± 3.4	<10^-6^
V10 (%)	2.6 ± 2.4	12.7 ± 4.3	<10^-6^
V5 (%)	4.0 ± 3.2	18.4 ± 5.3	<10^-6^
Dmean (Gy)	1.4 ± 0.9	5.2 ± 1.5	<10^-6^
Dmax (Gy)	39.7 ± 12.4	49.5 ± 1.7	<10^-5^

PTV, planning target volume; CTV, clinical target volume.

**Table 3:** Comparison of heart and left anterior descending coronary artery (LAD) dosimetric parameters in the two setup positions for the 17/41 patients with left breast cancer

	**Prone setup**	**Supine setup**	**p-value**
Heart (cc)	438.3 ± 79.5	465.3 ± 90.3	0.09
V20 (%)	1.5 ± 2.2	1.5 ± 1.5	0.90
V10 (%)	2.3 ± 2.8	2.6 ± 2.4	0.64
V5 (%)	4.0 ± 4.1	4.6 ± 3.9	0.59
Dmean (Gy)	1.9 ± 1.3	2.0 ± 1.0	0.95
Dmax (Gy)	36.8 ± 12.5	41.9 ± 9.8	0.06
Dmean LAD (Gy)	11.8 ± 9.8	12.0 ± 9.1	0.95
Dmax LAD (Gy)	27.8 ± 16.0	33.0 ± 13.2	0.14

Target coverage, assessed by V95, V105 and V107 of CTV and PTV, is reported in Table 2.

**Figure 2:**
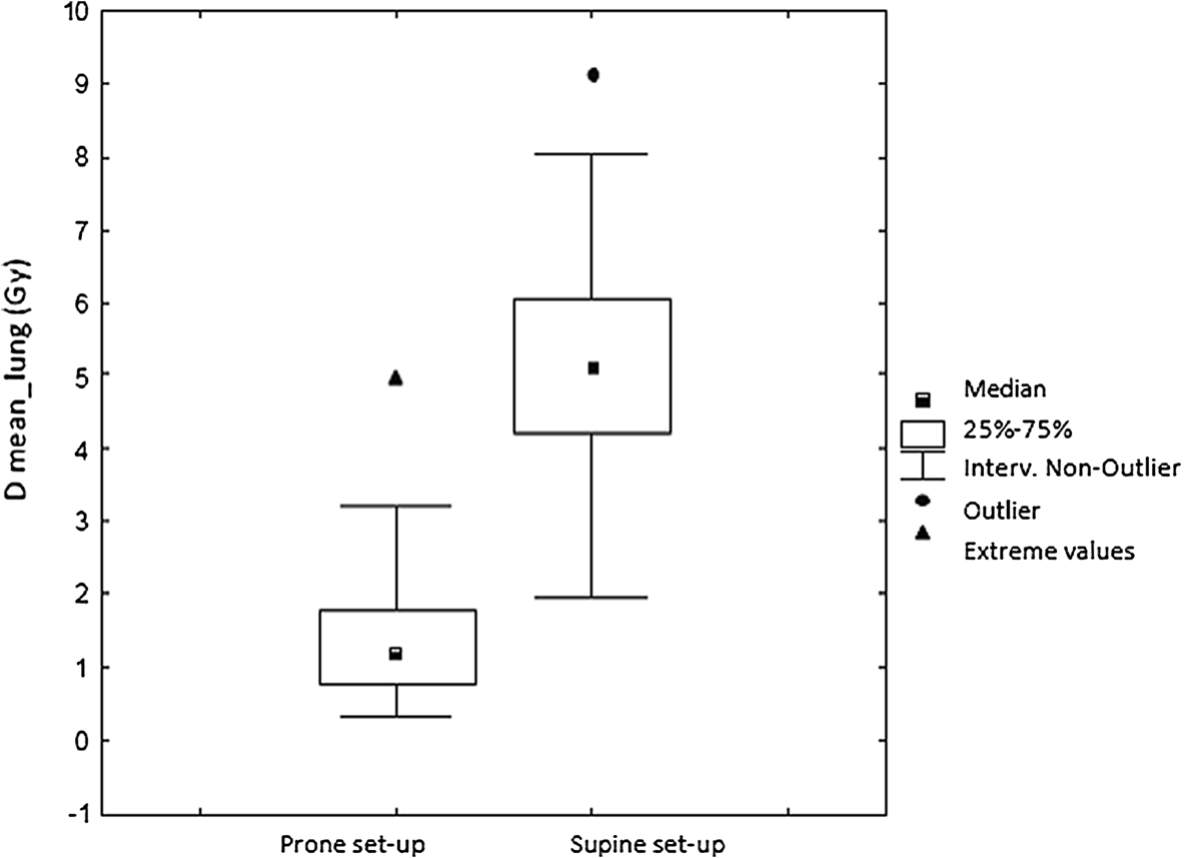
Lung mean dose in prone and supine setup.

The ipsilateral lung received less dose in prone than in supine position in all cases. The, V20, V10, and V5 as well as Dmean and Dmax resulted significantly lower in prone position (Table 2 and Figure 2).

A trend was evident in heart Dmax between the two setup positions in the 17 left sided breast cancers (Table 3). In 4/17 cases (23.5%), dose to the heart was higher in prone than in supine position. Dmax and Dmean to LAD in prone position resulted non-significantly lower in left sided breast cancers.

Considering target coverage and dose constraints to the healthy structures, 29 of the 41 patients (70.7%) included in the study were actually treated in prone setup because the treatment plan allowed a better sparing of lung and heart. Twelve patients (29.3%) were treated in supine position: 6 because of the inadequate target coverage in prone position in cases with extremely lateralized lesions, 2 because of the better dose homogeneity (smaller hot spots V105 and V107), 3 because of the lower dose to the heart in cases with medial lesions, and one because of the unfavorable geometry of treatment in prone position leading to a gantry versus breast board collision.

All the 29 patients treated with prone setup had an advantage in terms of dose to the lung (p<10^**-6**^ for all parameters) and the 10/17 patients (58.8%) with left-sided cancers had an advantage in all dosimetric parameters of the heart and the Dmean and Dmax of the LAD (Tables 3 and 4).

**Table 4:** Comparison of heart and left anterior descending coronary artery (LAD) coronary artery dosimetric parameters in the 10 patients with left breast cancer treated with in prone setup

	**Prone setup**	**Supine setup**	**p-value**
Heart (cc)	434.0 ± 92.6	469.3 ± 116.2	0.19
V20 (%)	0.6 ± 1.1	1.9 ± 1.6	0.026
V10 (%)	1.2 ± 1.8	3.1 ± 2.2	0.017
V5 (%)	2.4 ± 2.8	5.5 ± 4.3	0.005
Dmean (Gy)	1.6 ± 0.7	2.3 ± 1.0	0.9008
Dmax (Gy)	31.2 ± 14.0	41.9 ± 11.2	0.005
Dmean LAD (Gy)	9.4 ± 8.4	12.5 ± 9.5	0.03
Dmax LAD (Gy)	23.7 ± 15.2	34.5 ± 12.8	0.006

As far as the treatment reproducibility, data about the prone setup by using portal imaging checks showed median inter-fraction differences of 2.0 mm, 1.8 mm, and 2.5 mm in latero-lateral, longitudinal, and vertical axes respectively, similar to the data previously published for the supine position [6].

As far as acute toxicity, 18/41 of patients (43.9%) developed grade 1 and 19 (46.3%) developed grade 2 dermatitis consisting of moderate to brisk erythema, skin desquamation, typically located in the infra-mammary skin fold. Grade 3 acute dermatitis was observed in 4 patients (2.6%) with diffuse breast edema, pain and skin desquamation.

After 22.9 months mean follow-up (range: 12.1 - 52.3 months; median 17.5), late toxicity consisting of grade fibrosis 1 was found in 12/41 patients (29.3%). No significant difference was observed between the patients treated in prone and supine position either for acute or late toxicity. Looking at the occurrence of moist desquamation, there was a favorable trend (p=0.05) for patients treated in prone position.

## Discussion

The first study reporting a similar disease local control and toxicity in prone and supine treatment of breast with an evident reduction in lung and heart volumes inside treatment fields was conducted at Memorial Sloan Kettering Cancer Center in 1992 (2). The authors also demonstrated a reduction in dose inhomogeneity by approximately 15% compared with supine tangents in women with pendulous breasts. Subsequently, several studies showed advantages of the prone position include a more homogenous dose distribution with a reduction in the size of the hot spots, resulting in decreased acute reactions, irradiation of less normal tissue, and improved setup reproducibility [7-11]. A recent study showed that, the prone position was better than the supine position for sparing the lung and for sparing the heart in the majority of left breast cancer patients, especially in case of large breasts [12].

In our study, we analyzed women with smaller breasts and lower BMI compared to other US and North Europe series showing that prone position can be advantageous also in this group of patients and not only in case of very large breasts [11,13,14].

Some authors observed that in prone position, the better breast shape associated with lower thorax respiratory movements may reduce hot spots inside PTV with consequently better cosmetic result [2]. Six patients with extremely lateralized lesions had an inadequate coverage of PTV and CTV with prone setup, documented by V95, and consequently were treated in supine position. In this regard, other authors published data indicating a worst PTV coverage in extremely medially or laterally located lesions [15].

About lung reduction dose in prone setup, our data are consistent with those of other literature series [2-4,13]. In prone position in fact, lung parenchyma inside treatment fields is significantly lower, because of breast dislocation and substantially no influence of respiratory movements [8]. As a matter of fact, various authors demonstrated that the prone setup drastically reduces intra-fractional respiratory motion of the chest wall with good reproducibility, reporting a mean inter-fraction setup variability of less than 0.1 cm [8,10]. In our series, all patients showed a significant reduction in lung dose documented by V20, V10, V5, Dmean and Dmax. These data suggest that in case of lung as well as of heart diseases, prone setup could be considered regardless to the presence of pendulous breasts.

Literature data are not univocal about reduction in heart dose in prone setup. Some studies demonstrated a reduction in heart mean and maximum doses [2,4], others showed a higher mean dose to cardiac cavities [13] and some others did not find differences between prone and supine position [3,13,16]. These discordant data can be explained if it is considered that in prone position there is an anterior heart dislocation with a consequently differences in setup device and technical modalities of treatment as well as differences in cardiac cavities contouring and patients characteristics can influence the distribution of radiation dose [17].

From our analysis it emerged a non-statistically significant reduction in heart V5, V10, V20 and Dmax in prone set-up, but a trend for Dmean. On the contrary, in 23.5% of the patients with left sided cancer, the heart received a higher dose in supine setup. Other studies reported that in about 15% of left sided breast the prone setup did not present favorable dosimetry for heart parameters [18,19].

Because of its anatomical position, LAD is frequently inside treatment fields receiving a not negligible dose. Nowadays, there is not a reliable dose constraint for LAD, but there are literature studies that correlated dose to LAD with cardiac damage and risk of coronary disease [20]. Controversial opinions about the potential advantage of prone position for reducing the dose to the LAD were recently reported [18]. From our data, it emerged a non-significant reduction in mean and maximum dose in prone setup. Interestingly, Dmax to the LAD in patients with right sided breast cancer was relatively low as expected, but significantly lower in prone than in supine position.

Reproducibility of the prone versus supine setup position was quite similar in our experience. However, a specific training of the personnel involved is needed because longer time and more accurate checks are required in the first phase when the procedure is implemented. In our experience, the AlignRT system was of help in checking the right position of the patients either in supine or in prone setup. About patients compliance, some studies reported difficulty in prone positioning especially in elder women [7,9] but most authors observed that precision and reproducibility of prone setup was comparable to supine data [8,10,21]. In our study, only 3.6% of the patients was excluded because of low compliance to the prone setup.

Women with large and pendulous breasts can be technically challenging to treat with breast irradiation, resulting in high rates of severe acute dermatitis and late fibrosis that may cause unacceptable cosmetic outcome [22,23]. In the present study, we reported an acceptable rate of acute and late toxicity, similar to that reported in other literature studies [7,17].

Our study has some limitations such as the relatively small patients number related to the case selection focused only on pendulous breast patients, the availability of a CT-scan with small gantry size. Moreover, we did not consider the dose to contralateral breast; in this regard, other authors analyzed this parameter and failed to show any difference between the two setup positions [17].

## Conclusion

From our prospective study, the prone position allowed a significant decrease of ipsilateral lung dose in all patients and a favorable trend for heart dose in patients with left sided cancer that became significant in the group of patients actually treated with prone setup. Overall, the prone position offered a favorable alternative for irradiation of pendulous breasts in 71% of the cases considering our patient selection that excluded the largest breasts. In clinical practice, the choice of the treatment position should be based on an accurate analysis of each individual case.

## Abbreviations

RT: Radiotherapy; CTV: Clinical target volume; PTV: Planning target volume; LAD: Left anterior descending coronary artery; WBI: Whole-breast irradiation; BCS: Breast-conserving surgery; BMI: Body mass index; 3D-CRT: Three dimensional conformal radiotherapy; CT: Helical computed tomography; DVH: Dose volume histograms; Dmax: Maximum dose; Dmean: Mean dose.

## Competing interest

The authors declare they have no competing interests.

## Authors’ contributions

MK, LM, GG have made substantial contributions to conception and design the study, they have been involved in drafting the manuscript or revising it critically for important intellectual content; and have given final approval of the version to be published. TC, CP, GA, LD have made substantial contributions to acquisition of data, analysis and interpretation of data; they have been involved in drafting the manuscript. EN, MB have made substantial contributions to analysis and interpretation of data. All authors read and approved the final manuscript.
